# Fracture Toughness Behaviour of Nickel Alloy Steel 1.5662

**DOI:** 10.3390/ma17246117

**Published:** 2024-12-14

**Authors:** Nariman Afzali, Natalie Stranghöner, Peter Langenberg

**Affiliations:** 1 Institute for Metal and Lightweight Structures, University of Duisburg-Essen, Universitaetsstr. 15, 45141 Essen, Germany; natalie.stranghoener@uni-due.de; 2 IWT-Solutions AG, Mozartstraße 2A, 52064 Aachen, Germany; p.langenberg@iwt-ag.de

**Keywords:** nickel alloy steel, fracture toughness tests, Charpy-V impact tests, J_Ic_ and K_Jc_ evaluation, crack-tip opening displacement (CTOD)

## Abstract

Nickel significantly increases the toughness of steel and makes it ideal for use in applications that require high impact and fracture resistance at low temperatures. These inherent advantages position nickel steel as indispensable material in various domains, with a pronounced presence in stationary Liquefied Natural Gas (LNG) tanks and in the shipbuilding industry, particularly for tanks in vessels intended for the transport of liquefied ethane and LNG. The presented study focuses on assessing the fracture toughness behaviour of nickel alloy steel 1.5662+QT640 under sub-zero and cryogenic temperatures. The fracture performance of the material was evaluated, specifically emphasizing the impact toughness and fracture toughness characteristics of the material. Moreover, it was discussed if the transferability of the experimental results to the well-known fracture mechanics-based concept of EN 1993-1-10, which relies on the master curve concept, is possible. The results show that the master curve concept is not applicable to the nickel alloy steel 1.5662+QT640 due to its exceptional fracture toughness behaviour at very low temperatures.

## 1. Introduction

As global energy consumption continues to rise [1,2], there is growing interest in using gas, particularly natural gas, due to its lower CO_2_ emissions compared to coal and oil [3,4]. Due to the often large distances between the largest gas reserves and the main areas of consumption, the storage and transportation of large volumes of gas over long distances are necessary. Liquefying the gas offers a solution as it reduces its volume, simplifies storage and transportation and lowers costs. Ensuring the structural integrity of storage tanks under cryogenic temperatures is crucial for various container designs. Therefore, LNG storage tanks (tanks for Liquefied Natural Gas (LNG)) typically use materials that can withstand low temperatures, corresponding to the operating temperature of LNG.

The adoption of LNG as a maritime fuel demands careful consideration due to the risks associated with extremely low temperatures. This poses a significant risk of brittle fractures in the steel used for LNG tanks [5]. The danger is particularly acute for ships transporting LNG or involved in LNG bunkering, as unintended gas leaks can cause catastrophic structural damage [6]. To mitigate this hazard, it is crucial to use high-strength steels that can withstand cryogenic temperatures. These steels must endure long-term exposure to LNG flow and retain their ductility even at extreme low temperatures [5,7]. 1.5662+QT640 is a type of low-carbon, cold-resistant, weldable steel that is predominantly used in manufacturing LNG storage tanks. The nickel content is a crucial component of nickel alloy steel, enhancing its toughness properties [8,9].

Utilizing the potential of the higher material strengths of nickel alloy steels in accordance with EN 10028-4 [10] for the buckling safety verification of LNG tanks requires a detailed examination of the choice of material in order to avoid brittle fracture. Owing to the absence of more precise concepts [11], these aspects have traditionally been investigated using the Pellini approach, which necessitates a significant reduction in the yield strength of the steels for design purposes.

The Pellini concept, first introduced in the 1960s and rigorously validated in the petrochemical industry during the 1970s, focuses on determining the brittle fracture transition temperature, known as the nil ductility transition (NDT) temperature. This is measured using the drop weight test according to SEP 1325 [11] or ASTM E208 [12]. It demonstrates an empirical relationship between laboratory tests and component trials, defining brittle fracture failure in terms of the NDT temperature. However, this concept cannot be easily applied to modern designs or thicker wall sections today. Alternatively, a fracture mechanics-based concept in accordance with EN 1993-1-10 [13] offers a more accurate and efficient method for use in the construction industry, which is also the basis for the material selection concept for structural steels. The crack initiation concept enables a direct connection with simple laboratory tests such as the notched bar impact test and is established in standards such as EN 1993-1-10, EN 13445-2 [14] and EN 13480-2 [15].

The aim of the presented study was to investigate whether the principles of the fracture mechanics approach of EN 1993-1-10 can be applied to nickel alloy steels, as the fracture mechanics-based master curve approach has not yet been investigated for this type of steel.

The results presented in this contribution are the main outcomes within the scope of the research project 01IF21719N/P 1510 funded by the Federal Ministry of Economic Affairs and Climate Action as part of the “Industrial Collective Research” programme on the basis of a resolution of the German Bundestag. This project from the Research Association for steel Application (FOSTA), Düsseldorf, focuses on the “Load-bearing behaviour of meridionally and ring stiffened tanks and silos made of high strength (duplex) steels and low-temperature steels for pressure vessels”.

## 2. Experimental Investigation

### 2.1. Charpy-V Impact Tests

Compared to the fracture toughness test, the Charpy-V impact test is the simpler and therefore more cost-effective test method in terms of specimen preparation, test execution and determination of characteristic values. The disadvantage of the Charpy-V impact test is that it only characterizes the material, without considering the influence of the component geometry or providing any information about crack propagation. However, the Charpy-V impact test allows comparisons to be made between different materials, allowing a qualitative assessment of their toughness properties [16]. Nevertheless, to develop the master curve and evaluate the applicability of the concept to nickel alloy steels, conducting Charpy-V impact tests is essential.

The secondary objective of this study was to investigate how the material’s toughness properties are influenced by the thickness of the plate. The 1.5662+QT640 material, supplied by Universal Eisen und Stahl GmbH, Germany, was tested in two plate thicknesses, 15 mm and 25 mm, from one steel producer with one production lot for each investigated plate thickness. These plates underwent a quenching and tempering (QT) heat treatment. These thicknesses were selected to represent the typical range used in cryogenic applications, particularly in the construction of LNG storage tanks, which is the primary focus of this study. The chemical compositions of the investigated 1.5662+QT640 steels are presented in Table 1. These compositions conform to the specifications outlined in EN 10028-4, indicating that the steels meet the required standards for their intended use in LNG storage tanks (see Table 1).

**Figure 1:**
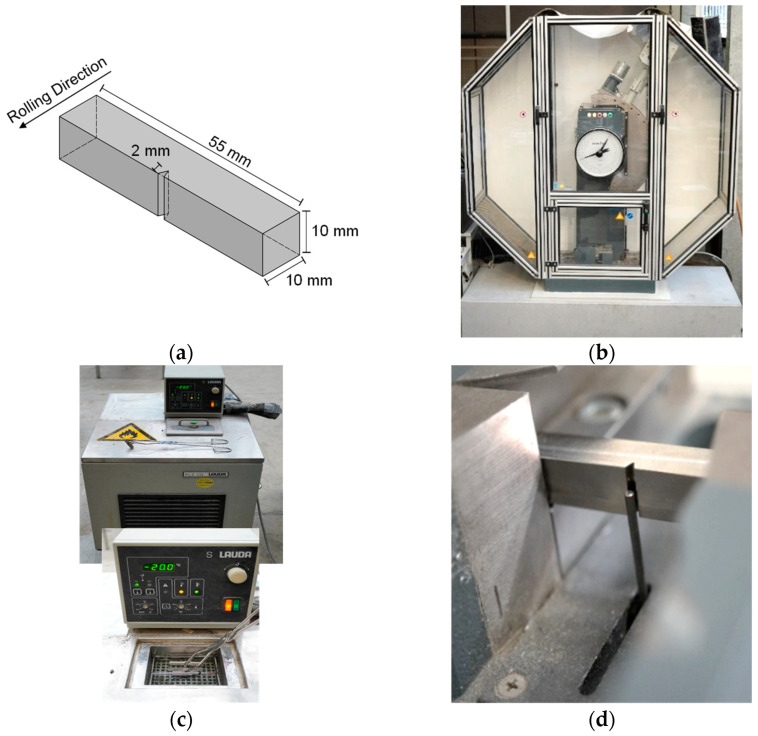
Test geometry and Charpy-V test setup: (**a**) geometry of the Charpy specimen; (**b**) testing machine (UDE); (**c**) specimen cooling using ethanol; and (**d**) specimen position in the machine.

A total of 54 Charpy-V impact tests were conducted in accordance with EN ISO 148-1 [17]. All test specimens were prepared in line with EN ISO 148-1, featuring a cross-section of 10 mm × 10 mm and a length of 55 mm. The notch was designed in a V-shape in the middle of the length, with a notch angle of 45°, a notch radius of 0.25 mm, and a notch depth of 2 mm, as shown in Figure 1a. The Charpy-V impact specimen was oriented transverse to the rolling direction (TD) with the notch machined along the rolling direction aligned with the elongated microstructure of the plate (T-L orientation). This orientation of the Charpy-V impact specimen was chosen because it is more critical and absorbs less energy as transverse loading stresses the grain boundaries, which are weaker and more prone to fracture, resulting in less energy absorption.

The Charpy-V tests were performed using a Charpy 450 J pendulum impact testing machine from Zwick/Roell at the Institute for Metal and Lightweight Construction at the University of Duisburg-Essen, as depicted in Figure 1b–d. The notched samples were struck by a controlled-weight pendulum, swung from a fixed height, and the work absorbed by the specimen was measured from this test [17].

The aim of this study was to fully characterize the temperature-dependent behaviour of the material, particularly in the ductile, to a brittle transition temperature range and to determine the transition temperatures.

A series of at least 21 specimens was prepared for each plate thickness to be tested at different temperatures between room temperature and about −196 °C, so that the impact energy was determined for at least seven temperatures, with three tests per temperature to establish a transition temperature curve. During the test, the Charpy-V specimens were positioned in the centre of the supports in the pendulum impact machine so that the notch was exactly opposite the point of impact of the pendulum hammer on the specimen (see Figure 1d).

**Figure 2:**
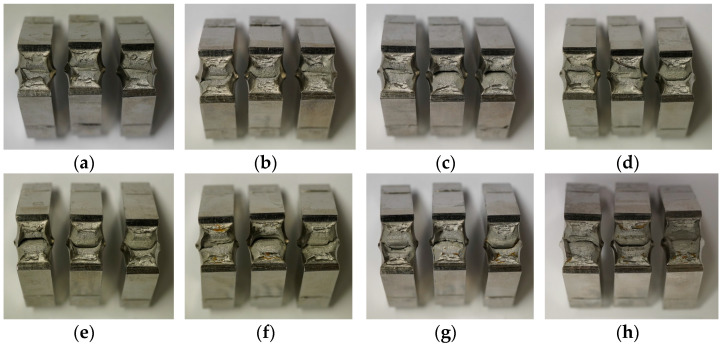
Exemplary photos of fractured surfaces of Charpy-V test specimens of 1.5662+QT640, 25 mm: (**a**) 25 °C; (**b**) 0 °C; (**c**) −20 °C; (**d**) −50 °C; (**e**) −80 °C; (**f**) −110 °C; (**g**) −150 °C; and (**h**) −196 °C.

The fracture surface images of the 1.5662+QT640 base material specimens made from the 25 mm thick plate are shown in Figure 2. All notch impact energy values are presented in Table 2.

To evaluate the transition curve from the experimental results, an approximation was made using a hyperbolic tangent function based on the Oldfield regression model [18], as shown in Equation (1). Where CVE is the Charpy impact energy in Joules, T is the temperature in °C and A, B and C are fitting constants of the hyperbolic tangent function.

(1) $$CVE\left(T\right)=A\cdot \left[1+tanh(\frac{T-B}{C})\right]$$

**Figure 3:**
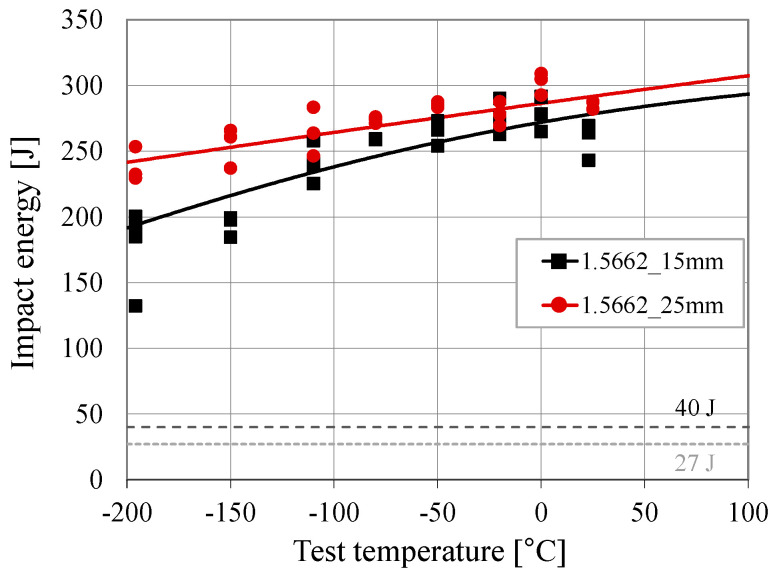
Impact toughness transition curves based on experimental tests.

All the impact energy transition curves are shown in Figure 3 to illustrate the effect of the plate thickness on the transition curves. It becomes obvious that, based on the obtained fitted curves, it is not possible to identify the relevant transition temperature criteria (such as T_27J_ or T_40J_) for this material.

It can be seen from Figure 3 and Table 2 that the mean values of the absorbed energy at room temperature are 261 J for the specimens made of the 15 mm plate and a fairly similar value of approximately 285 J for the specimens made of the 25 mm plate. However, this difference is somewhat more pronounced at the lowest temperature (−196 °C) where the mean values of the absorbed energy are 178 J for the specimens made of the 15 mm plate and 238 J for the specimens made of the 25 mm plate. Both plates with thicknesses of 15 mm and 25 mm were produced by the same steel producer. Herewith, it could be expected that the absorbed energy decreases with increasing plate thickness, exemplarily shown for duplex stainless steels in [19]. This was not observed in the presented investigations. The observed behaviour might be explained by several factors: (1) potential variations during the plate production, e.g., in the rolling or heat treatment processes, might lead to slight differences in the microstructural characteristics of the two different production lots, i.e., different grain sizes that influence the potential of energy absorption. Due to the limitations of the current project, neither a comprehensive microstructural investigation including grain size analysis was feasible, nor further plates from different steel producers in different plate thicknesses could be investigated. However, it is highly recommended that such studies will be carried out in the future to gain a deeper insight into the fracture toughness behaviour of nickel alloy steels. Nevertheless, it should be noted that the absorbed energies for both investigated plate thicknesses are well above the minimum impact energy values specified in EN 10028-4 for this steel grade (see Table 2). Herewith, the requirements of EN 10028-4 are fulfilled for both plate thicknesses.

Although the nickel alloy steel 1.5662+QT640 does not show any ductile to brittle temperature behaviour, the absorbed energy tends to decrease with decreasing temperature. However, even at the lowest utilized temperature of −196 °C (liquid nitrogen), the material does not exhibit any brittle behaviour (see Figure 2h).

### 2.2. Fracture Toughness Tests

**Figure 4:**
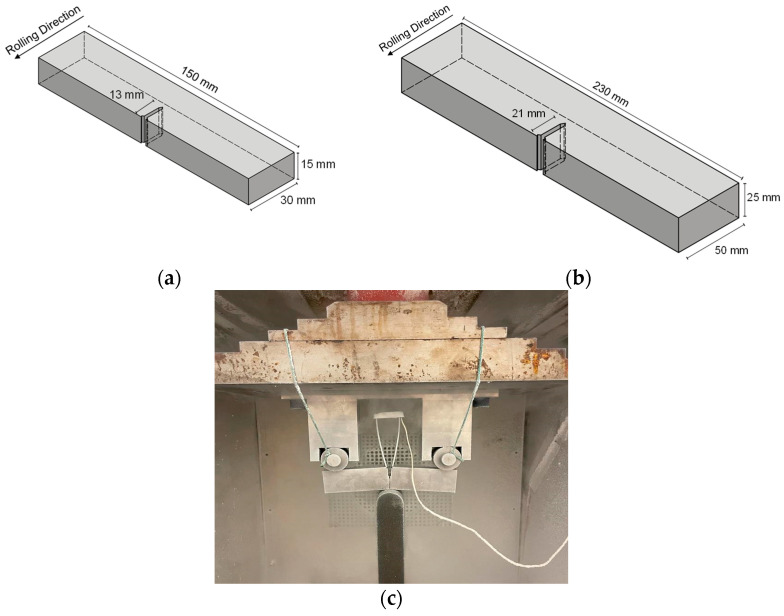
Specimen geometry and test setup for fracture toughness testing: (**a**) made from 15 mm thick plate; (**b**) made from 25 mm thick plate; and (**c**) fracture toughness test setup.

Charpy impact tests are not capable of capturing the fracture behaviour of the investigated components. To gain a better understanding of the fracture toughness behaviour, including resistance to crack growth, fracture toughness tests were performed as a rational consequence of obtaining more precise and transferable information about the material. The tests were performed according to ASTM E1820 [20] using SENB (Single Edge Notched Bend) specimens fabricated from nickel alloy steel 1.5662+QT640 and plate thicknesses of 15 mm and 25 mm. The dimensions were 25 mm × 50 mm × 230 mm and 15 mm × 30 mm × 150 mm, respectively, derived according to ASTM E1820 [20] (see Figure 4a,b). The SENB specimens were taken transverse to the rolling direction of the plates with notches machined longitudinally to the rolling direction (along the elongated microstructure of the material). The pre-fatigue crack length was determined for each specimen at room temperature, resulting in an a_0_/W value of 0.5, where a_0_ is the original crack length, and W is the width of the specimen. In order to obtain a straight crack front, lateral grooves with a depth of 1% of the plate thickness were machined on both sides of the specimen at an angle of approximately 45° after pre-fatigue. These side grooves also ensured the highest stress (plane strain) over the entire specimen, even on the flanks where plane stresses would normally dominate. For this study, the fracture toughness evaluation of the test results included the J-integral (J_Ic_), K_Jc_ and calculated crack tip opening displacement (CTOD) values based on ASTM E1820 [20]. All three values were determined simultaneously for each test.

The fracture toughness tests were performed using a 100 kN hydraulic testing machine with a three-point bending test stand and a cooling chamber, as shown in Figure 4c. A test environment temperature of −150 °C to −160 °C was achieved using liquid nitrogen in a controlled cooling chamber. The test temperature was maintained with a high accuracy of ±0.2 °C throughout the testing. The minimum time that the specimen was held at test temperature was determined by ASTM E1820 based on the plate thickness to ensure a uniform temperature distribution throughout the specimen prior to testing. For both plate thicknesses, the SENB samples were held at the test temperature for a minimum of 30 min prior to testing to achieve thermal equilibrium.

**Figure 5:**
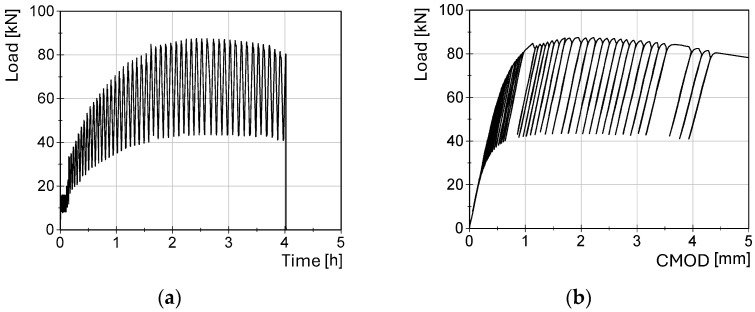
Loading/unloading protocol for fracture toughness test on specimen made from 25 mm thick plate at −160 °C: (**a**) loading/unloading cycles; and (**b**) load-CMOD diagram.

The load was applied in a displacement-controlled manner with a constant rate of 0.01 mm/s. The crack mouth opening displacement (CMOD) was monitored using a clip gauge. The fracture toughness tests were performed using the compliance method, which consists of several loading and unloading cycles, with an unloading ratio set at 50% of the actual maximum load [21] (see Figure 5).

**Figure 6:**
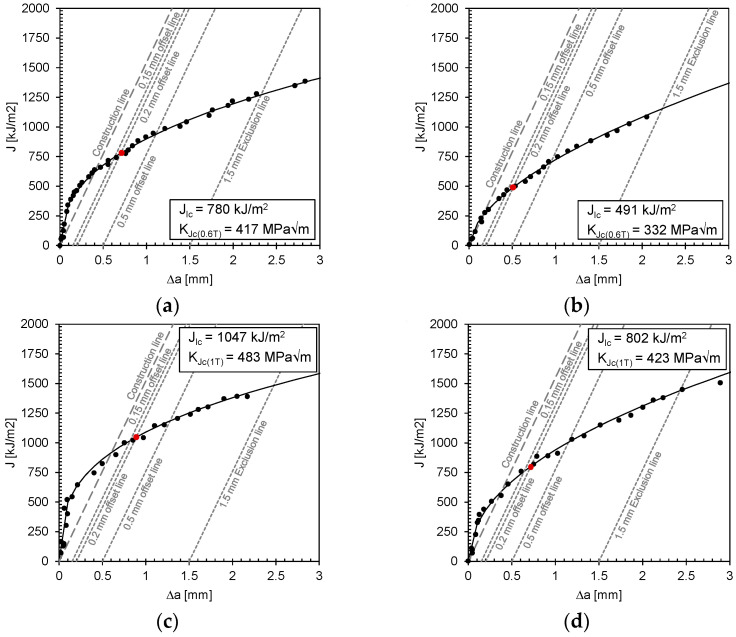
J-Δa curves and calculated K_Jc_ values for 1.5662 specimens: (**a**) 15 mm plate tested at −150 °C; (**b**) 15 mm plate tested at −160 °C; (**c**) 25 mm plate tested at −150 °C; and (**d**) 25 mm plate tested at −160 °C.

Figure 6 shows J-Δa curves for 1.5662+QT640 specimens made from 15 mm and 25 mm thick plates tested at −150 °C and −160 °C. In these curves, “J” is the fracture energy per unit area of fracture, and “Δa” is the crack propagation in millimetres. The construction line was drawn based on Equations (2) and (3) where σ_YS_ is the yield strength, and σ_TS_ is the ultimate strength of the material, both in MPa.

(2) $$J=2\sigma_{Y}∆a$$

(3) $$\sigma_{Y}=\frac{\sigma_{YS}+\sigma_{TS}}{2}$$

For the evaluation of the fracture toughness tests, the determination of the mechanical properties of the material at each test temperature is essential. However, no tensile tests could be carried out at cryogenic temperatures. For this reason, the mechanical properties at each test temperature were calculated considering the room temperature results and the cubic polynomial equation derived from the available experimental data by Anoop et al. [22] (see Table 3). Based on the estimated mechanical properties of the material at test temperature, the construction line for the J-Δa curve could be drawn. J_Ic_ was then determined by fitting a power law to the data points within the range of a 0.15 mm to 1.5 mm offset line parallel to the construction line, as shown in Figure 6 and described by Equations (4), where C_1_ and C_2_ represent the power law coefficients.

In Figure 6, J_Ic_ is defined as the intersection of the fitted curve with the 0.2 mm offset line. K_Jc_ was then calculated using Equation (5), where E is the modulus of elasticity at the test temperature calculated according to Anoop et al. 2021 [22]. The Poisson’s ratio ν was set to 0.3. The size correction of K_Jc_ data was also performed on 15 mm thick test specimens in accordance with ASTM E1921 [23]. According to this standard, when data are obtained from specimens of a size other than 1T, all K_Jc_ values must be converted to the 1T size equivalent using Equation (6). K_Jc(x)_ is the K_Jc_ value for a specimen size B_x_, where B_x_ is the gross thickness of the prediction with side grooves ignored and K_Jc(o)_ is the K_Jc_ value for a specimen size B_o_, where B_o_ is the gross thickness of the test specimens with side grooves ignored.

(4) $$J=C_{1}{\left(\frac{\Delta a}{k}\right)}^{C_{2}}$$

(5) $$K_{Jc}=\sqrt{\frac{EJ_{Ic}}{\left(1-\vartheta^{2}\right)}}$$

(6) $$K_{Jc(x)}=20+[K_{Jc\left(o\right)}-20]{\left(\frac{B_{o}}{B_{x}}\right)}^{\frac{1}{4}}$$

**Figure 7:**
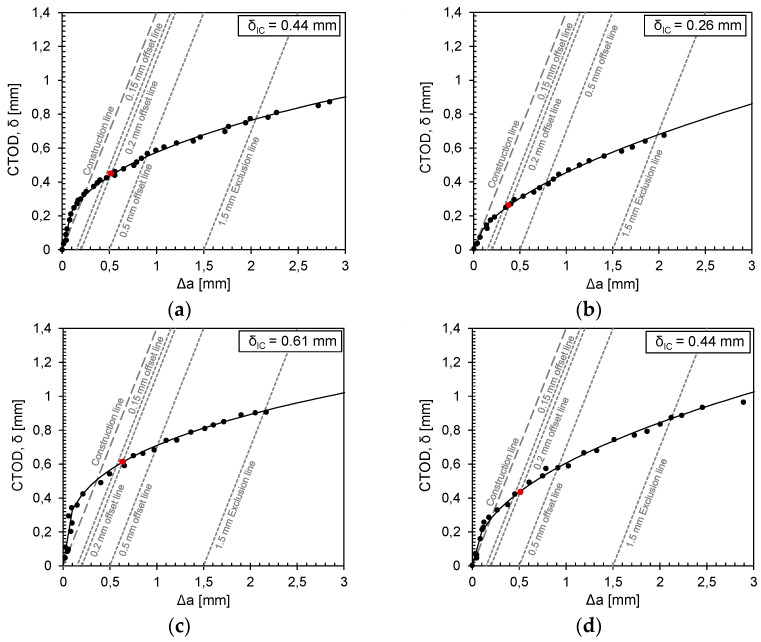
Calculated CTOD-Δa curves for 1.5662+QT640 specimens: (**a**) 15 mm plate tested at −150 °C; (**b**) 15 mm plate tested at −160 °C; (**c**) 25mm plate tested at −150 °C; and (**d**) 25mm plate tested at −160 °C.

**Figure 8:**
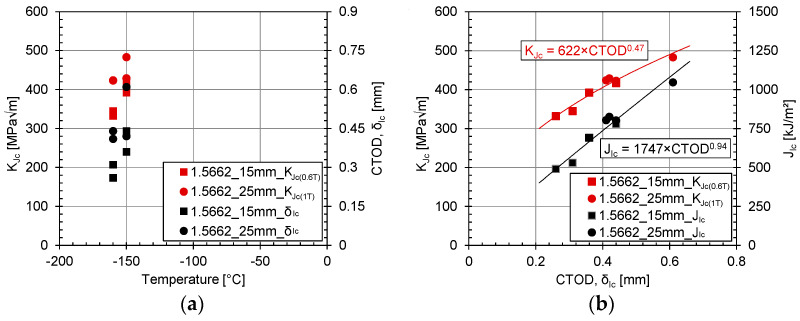
Overview on fracture toughness test results: (**a**) K_Jc_ vs. temperature and δ_Ic_ vs. temperature; and (**b**) K_Jc_ vs. δ_Ic_ and J_Ic_ vs. δ_Ic_.

The calculation of CTOD for the resistance curve at each point along the force–displacement curve was derived using Equation (7). The variable “m” was determined by considering the mechanical properties of the material, such as yield strength (σ_YS_) and ultimate strength (σ_TS_), as well as the actual crack size. δ_Ic_ is identified as the intersection of the fitted curve with the 0.2 mm offset line (see Figure 7). Table 4. shows the values for CTOD (δ_Ic_), J_Ic_, K_Jc(1T)_ and K_Jc(0.6T)_. Furthermore, Figure 8a gives an overview of the fracture toughness test results J_Ic_ and the derived CTOD values.

(7) $$\delta_{i}=\frac{J_{i}}{m_{i}\times \sigma_{Y}}$$

Finally, Figure 8b shows two derived correlations for the investigated nickel alloy steel 1.5662+QT640, illustrating the correlation between CTOD and K_Jc_ (see Equation (8)) and CTOD and J_Ic_ (see Equation (9)). The equations given can be considered as preliminary correlation equations, for which the following two important considerations must be emphasized:

Firstly, the CTOD values are derived from the J values, which means that factors such as ‘m’ and the yield strength also influence the correlation, and changes in these values may affect the correlation fit. Secondly, it must be recognized that the number of test results available is not sufficient to make a conclusive statement about the correlation between the fracture toughness values for the investigated steel grade.

The results show the direct influence of the temperature on the resulting fracture toughness. As expected, both the K_Jc_ and CTOD values show a decrease in the achieved toughness with a decreasing test temperature (see Table 4, Figure 6 and Figure 7).

In the past, the influence of the plate thickness on the fracture toughness of the material was investigated for both carbon steels and duplex stainless steels and was documented by various researchers, including [19,24]. It is important to note that the current study only includes two different plate thicknesses from different batches, so no final conclusion can be drawn in this case. However, in the presented investigation, it appears that the specimens with the greater plate thickness of (25 mm) show higher fracture toughness values than those specimens made from the thinner plates (15 mm). This unexpected outcome also becomes obvious for the Charpy-V impact tests conducted at very low temperatures (but still within the upper shelf of the transition curve), as shown in Figure 3. To gain a clearer understanding of the effect of the plate thickness on the fracture toughness behaviour of nickel alloy steel, an extended test programme is recommended covering various plate thicknesses, material producers and test temperatures to check and probably improve the developed correlations.

(8) $$K_{Jc}=622\times {CTOD}^{0.47}$$

(9) $$J_{Ic}=1747\times {CTOD}^{0.94}$$

**Figure 9:**
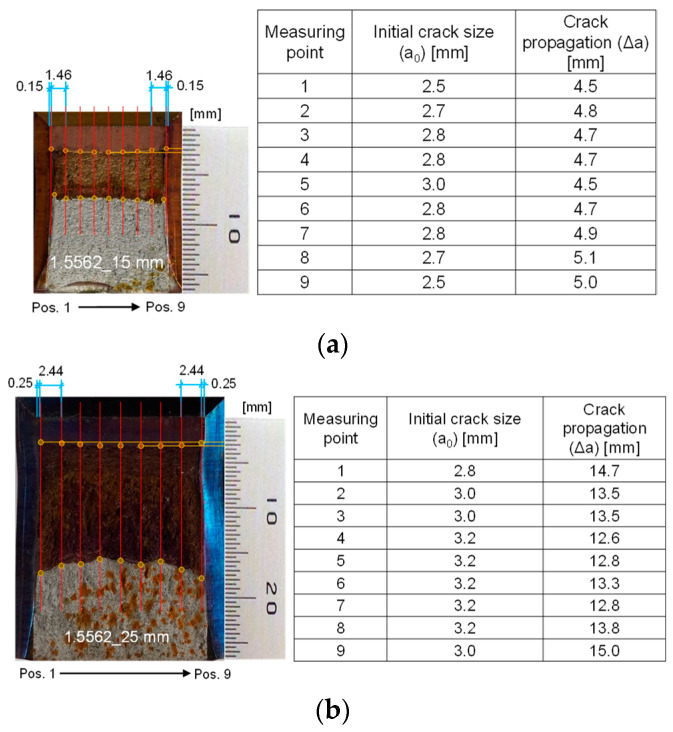
Examples of fracture surfaces after fracture toughness testing: (**a**) 15 mm plate tested at −150 °C; and (**b**) 25mm plate tested at −160 °C.

To validate these tests according to ASTM E1820, all fracture surfaces were analyzed (see Figure 9). Nine initial crack size measurements were taken for each specimen. The results show that all tests are valid as none of the nine initial crack sizes and final physical crack size measurements deviated by more than 0.1 (b_o_B_N_)1/2 from the mean physical crack size according to ASTM E1820 (with the following: b_0_ is the remaining specimen width (b_0_ = W − a_0_), a_0_ is the initial crack size, W is the initial specimen width and B_N_ is the net specimen thickness).

As part of the presented investigation, an attempt was made to calculate the reference temperature (T_100_) in accordance with ASTM E1921. Hereby, it must be considered that the K_Jc_ values determined in these experimental investigations are significantly higher than the K_Jc_ limit specified in ASTM E1921, according to Equation (10).

The K_Jc_ limit is approximately 256 MPa√m for the specimens made from 15 mm plates and approximately 340 MPa√m for the specimens made from 25 mm plates at temperatures between −150 °C and −160 °C. Herewith, the achieved K_Jc_ values were considerably higher than the specified K_Jc_ limits for all tests performed (see Table 4).

(10) $$K_{Jclimit}=\sqrt{\frac{{Eb}_{0}\times \sigma_{YS}}{30(1-\upsilon^{2})}}$$

According to ASTM E1921, if the K_Jc_ limit is exceeded, the values must be censored, which includes all obtained results. Therefore, in the absence of the transition temperatures T_27J_ or T_40J_ or the reference temperature T_100_, it is not possible to assess the applicability of the current correlation between transition and reference temperatures used in the EN 1993-1-10 concept. Herewith, the master curve concept [16,25,26,27] applied in EN 1993-1-10 cannot be applied to the investigated 1.5662+QT640 nickel alloy steel due to its extremely good low-temperature fracture toughness behaviour.

## 3. Conclusions

Nickel alloy steel 1.5662 is particularly suitable for cryogenic applications. It can be used at temperatures as low as −196 °C and has high fracture toughness properties. However, a comprehensive investigation of the fracture properties of this type of steel was carried out in order to investigate whether the master curve concept included in the fracture mechanics concept of EN 1993-1-10 for the choice of steel material to avoid brittle fracture can be applied to nickel alloyed steels as well. Up to now, the fracture mechanics master curve concept has not been investigated for very low-temperature pressure vessel steels.

A total of 54 Charpy V-notch impact tests according to EN ISO 148-1 were performed on the low-temperature pressure vessel steel 1.5662+QT640 according to EN 10028-4 for two different plate thicknesses (15 mm and 25 mm) in order to cover the influence of the material thickness. The following results could be observed:

- All tested specimens exhibited ductile fracture, even at the extremely low temperature of −196 °C, so that it was not possible to determine the known transition temperature criteria for ferritic steels such as T_27J_ or T_40J_.
- However, a slight decrease in the absorbed energy could be observed with decreasing temperature.
- At room temperature, the specimens of both plate thicknesses showed approximately the same amount of absorbed energy, but, at the lowest tested temperature of −196 °C, they showed a rather clear difference with slightly higher absorbed energies for the thicker plate thickness of 25 mm. This difference may be due to potential variations in the plate production process, such as differences in rolling or heat treatment, which may result in subtle microstructural differences between the two production lots.

Furthermore, fracture toughness tests were performed and evaluated in accordance with ASTM E1820. Standard SENB specimens were selected to analyze the crack growth resistance for specimens made of both plate thicknesses of 15 mm and 25 mm. The fracture toughness evaluation included J_Ic_, K_Jc_ and calculated CTOD (δ_Ic_) values in accordance with ASTM E1820 for all tested specimens. The following results could be observed:

- It appears that the SENB specimens made from 25 mm plates show slightly higher fracture toughness values than those made from 15 mm plates. This difference may be due to the different conditions under which the two plate thicknesses were produced and cannot be interpreted as a general trend.
- As one result, two correlation equations could be derived from the fracture toughness test data for the tested nickel alloy steel 1.5662+QT640 to link CTOD and K_Jc_ values and CTOD and J_Ic_ values.
- It could be shown that, in the absence of the usually applied transition temperatures T27J or T40J and the reference temperature T100, the master curve concept as the basis of EN 1993-1-10 cannot be applied to nickel alloy steel 1.5662+QT640 due to its extremely good low-temperature fracture toughness behaviour.

## Figures and Tables

**Table 1:** Chemical composition of 1.5662+QT640 according to material certificate and EN 10028-4.

Material	Plate Thickness	Element						
		C	Si	Mn	P	Cr	Mo	Ni
1.5662+QT640	15 mm	0.04	0.24	0.53	0.009	0.06	0.004	8.95
	25 mm	0.04	0.23	0.51	0.006	0.04	0.01	9.05
	-	≤ 0.10 ^1^	≤ 0.35 ^1^	0.30–0.80 ^1^	≤ 0.020 ^1^	-	≤0.10 ^1^	8.5–10 ^1^

^1^ According to EN 10028-4.

**Table 2:** Energy absorption values obtained from Charpy-V impact tests and minimum values of the impact energy according to EN 10028-4.

Material	Plate Thickness [mm]	Temperature [°C]							
		RT	0	−20	−50	−80	−110	−150	−196
		Impact Energy [J] (Mean Values)							
1.5662+QT640	15	261	281	275	267	259	241	194	178
	25	285	302	278	285	274	264	245	238
	5 to 70 ^1^	70 ^1^	70 ^1^	70 ^1^	70 ^1^	70 ^1^	-	50 ^1^	40 ^1^

^1^ According to EN 10028-4.

**Table 3:** Calculated mechanical properties of the material at low temperatures based on Anoop et al. [22].

Material	Plate Thickness	Temperature [°C]	f_y_ [MPa]	f_u_ [MPa]	E [MPa]
1.5662+QT640	15 mm	RT ^1^	696 ^2^	738 ^2^	180.716 ^2^
		−150	787	970	202.724
		−160	798	986	203.037
	25 mm	RT ^1^	670 ^2^	712 ^2^	195.296 ^2^
		−150	761	944	188.814
		−160	772	960	189.146

^1^ Room temperature, ^2^ test performed at the Institute for Metal and Lightweight Structures, University of Duisburg-Essen at room temperature.

**Table 4:** Fracture toughness test results.

Material	Plate Thickness	Temperature [°C]	J_Ic_ [kJ/mm²]	K_Jc(1T)_/ K_Jc(0.6T)_ [MPa√m]	CTOD, δ ^1^ [mm]
1.5662+QT640	15 mm	−150	691	347/392	0.36
			780	369/417	0.44
		−160	491	295/332	0.26
			531	306/345	0.31
	25 mm	−150	1047	483/-	0.61
			826	429/-	0.42
		−160	802	423/-	0.44
			804	424/-	0.41

^1^ Calculated values, see Figure 7 and Equation (7).

## Data Availability

The data are presented in this article.
